# Effects of Urinary Incontinence Subtypes on Quality of Life and Sexual Function among Women Seeking Weight Loss

**DOI:** 10.1007/s00192-024-05977-z

**Published:** 2024-11-21

**Authors:** Zhao Tian, Linru Fu, Xiuqi Wang, Tangdi Lin, Wei Chen, Zhijing Sun

**Affiliations:** 1 Department of Obstetrics and Gynecology, Peking Union Medical College Hospital, Peking Union Medical College, Chinese Academy of Medical Sciences, National Clinical Research Center for Obstetric & Gynecologic Diseases, No. 1 Shuaifu Road, Dongcheng District, Beijing, People’s Republic of China 100730; 2 Department of Clinical Nutrition, Peking Union Medical College Hospital, Peking Union Medical College, Chinese Academy of Medical Sciences, Beijing, China

**Keywords:** Urinary incontinence, Quality of life, Obesity

## Abstract

**Introduction and Hypothesis:**

The objective was to detect subtypes of urinary incontinence (UI) and their effects on quality of life (QoL) and sexual function among women seeking weight loss.

**Methods:**

A cross-sectional study focusing on women seeking weight loss with UI symptoms was carried out. Participants were stratified into three groups: stress UI, urgency UI, and mixed UI groups. The effects of the three groups on QoL and sexual function were compared.

**Results:**

A total of 564 individuals (46.8%) were reported to present with UI symptoms. Among these, 216 (38.3%), 71 (12.6%), and 277 (49.1%) had stress UI, urgency UI, and mixed UI respectively. The severity of UI was greater in the urgency UI and mixed UI groups than in the stress UI group, with varying ratios observed among patients with different severities of UI: stress UI was highest in mild cases, and the mixed UI was highest in moderate or severe cases. Mixed UI had the most detrimental effect on QoL and sexual function. However, after controlling for the severity of UI, mixed UI still had a greater detrimental effect on UI-specific QoL, and no differences were identified among the three groups regarding general QoL or sexual function.

**Conclusion:**

This study revealed variations in the constituent ratios of UI subtypes related to the severity of UI and the effects of various UI subtypes on QoL and sexual function among women seeking weight loss. Notably, the mixed UI demonstrated the most severe symptoms and the most detrimental impact, particularly as assessed by UI-specific QoL questionnaires.

**Supplementary Information:**

The online version contains supplementary material available at 10.1007/s00192-024-05977-z.

## Introduction

Pelvic floor dysfunction (PFD), recognized as one of the five major chronic diseases affecting women’s quality of life (QoL), poses a significant health challenge for contemporary women. Among the various subtypes of PFD, urinary incontinence (UI) is particularly prevalent. The occurrence of PFD is thought to be associated with pregnancy and delivery, aging, menopause, increased abdominal pressure, etc. [1, 2]. Many studies have shed light on the correlation between obesity and PFD [3, 4], especially UI.

Obesity has also become a prevalent global health issue, with an estimated 1 in 5 adults predicted to have obesity by the year 2025 [4]. Excessive weight exerts additional stress on the pelvic floor, leading to structural modifications and functional abnormalities [3]. A considerable number of previous studies [3, 5, 6] have reported a notable increase in the prevalence of UI in individuals with overweight or obesity, particularly those with central obesity. One meta-analysis [5] involving 29,618 individuals concluded that there is an increased risk of UI in middle-aged and elderly women with overweight (odds ratio of 1.27) and obesity (odds ratio of 1.60).

The subtypes of UI include stress UI, urgency UI, and mixed UI. A nationwide epidemiological survey in 2009 [7] revealed that the overall prevalence rate of UI among Chinese adult women was 30.9%, with a corresponding distribution of 61% for stress UI, 31% for mixed UI, and 8% for urgency UI. Another nationwide epidemiological survey in 2022 [8] revealed that the standardized incidences of UI, stress UI, urgency UI, and mixed UI were 21.2, 13.1, 3.0, and 5.1 per 1,000 person-years respectively. Previous studies have demonstrated that different subtypes of UI may have varying degrees of impact on QoL and sexual function [9–11]. Compared with stress UI, mixed UI, and urgency UI may have more severe impacts on QoL, but their effects on sexual function remain controversial according to current research [9–11]. Moreover, there is currently limited research on the detailed subtypes of UI and their impact on QoL and sexual function among women with overweight or obesity. A previous Brazilian study [12] included 221 participants with obesity, among whom 118 (53.4%) reported UI episodes: mixed UI, stress UI, and urgency UI were reported by 52.5%, 33.9%, and 13.5% respectively. However, the limited sample size and failure to consider confounding factors such as the severity of UI greatly affected the credibility of the conclusions.

Current guidelines recommend multidisciplinary interventions, mainly including supervised pelvic floor training and weight loss management for women with both UI and obesity [4, 13]. Given that comprehending the distinctive effects of UI subtypes on patients’ QoL and sexual function is pivotal for devising personalized interventions and prevention strategies, thereby aiding in the development of more tailored management approaches, this study sought to examine the composition ratio of distinct subtypes of UI and to assess their effects on QoL and sexual function among individuals with both overweight or obesity and UI.

## Materials and Methods

### Recruitment and Data Collection

An online survey targeting women seeking weight loss was carried out from September 2023 to January 2024 within the clinical nutrition department of our center. For those with UI, additional comprehensive datasets were gathered, which included essential demographic information and validated questionnaires of the Chinese version including the International Consultation on Incontinence Modular Questionnaire-Urinary Incontinence Short Form (ICIQ-UI-SF) [14], the Incontinence Impact Questionnaire 7 (IIQ-7) [15, 16], the Urogenital Distress Inventory 6 (UDI-6) [16], the European Quality of Life-5 Dimensions 5-Level (EQ-5D-5L) [17], and the Short-form Prolapse Incontinence Sexual Questionnaire (PISQ-12) [18]. To enhance the accuracy of data collection, several measures were implemented. Clear and detailed guidelines were provided during survey-filling and logical checks were built into the questionnaire design to automatically flag any inconsistent or implausible responses. Surveys were administered through official hospital channels, thereby improving the engagement of respondents. Moreover, demographic information provided by participants was cross-verified with medical records to ensure the accuracy and reliability of the data collected.

The inclusion criteria were individuals who had UI symptoms, were at least 18 years old, and had a body mass index (BMI) of 25 kg/m^2^ or more. Conversely, participants who met any of the following criteria were excluded: previous treatment for pelvic floor disorder, pregnancy or childbirth within the past 6 months, prolapse extending beyond the hymenal level, urinary tract infection or gynecological reproductive system infection in the last month, severe systemic disease, pelvic or abdominal malignancy, or a history of bariatric surgery or pharmaceutical weight loss interventions.

### Details of the Included Questionnaires

#### Assessment of Urinary Incontinence

The definitions of UI and stress UI subtypes used in this study adhere to International Continence Society guidelines [13]. UI symptoms were defined as complaints of involuntary loss of urine. Stress UI was defined as a complaint of involuntary loss of urine caused by physical exertion (e.g., sporting activities), or sneezing or coughing, but no urgency UI symptoms. Urgency UI was defined either as an urge to urinate but unable to reach the toilet before leaking or having a strong sudden urge to go to the toilet to urinate with no advance warning, but no stress UI symptoms. Mixed UI symptoms were defined as at least one stress and one urge symptom. The severity of UI was assessed via the ICIQ-UI-SF [14], whose overall score, which is based on the first three items (0–21), classifies severity as mild (0–7), moderate (8–13), or severe (14–21).

#### Quality of Life

The specific QoL of patients with UI was evaluated using the IIQ-7, which comprises seven questions rated 0–3 points each, totaling 0–21 points, and the UDI-6, which comprises six questions rated 0–3 points each, totaling 0–18 points; higher scores suggest a stronger impact. The general QoL is additionally assessed via the EQ-5D-5L, which measures mobility, self-care, usual activities, pain/discomfort, and anxiety/depression (1–5 score range per dimension) and provides an overall health self-evaluation via the associated visual analog scale (EQ-VAS, 0–100 score range).

#### Sexual Function

The PISQ-12 was used to investigate sexual function, grouped into behavioral/emotive (items 1-4), physical (items 5-9), and partner-related (items 10-12) domains. Scores are calculated by adding up scores of each question (0 = always, 4 = never), with items 1-4 reverse.

### Sample Size

The primary outcome of this study was the QoL among different UI subtypes measured by UDI-6 among women seeking weight loss. Owing to a lack of prior research on this topic, we conducted a pilot study on 100 patients. The initial analysis of participants with stress UI (*n* = 35), urgency UI (*n* = 12), and mixed UI (*n* = 53) revealed that the mean ± standard deviation of UDI-6 scores were 2.6 ± 2.1, 2.2 ± 2.1, and 4.2 ± 3.3 respectively. A one-way ANOVA sample size calculation using PASS 15 software indicated that each group should have at least 63 participants for a two-sided alpha level of 0.05 and 90% power. Accounting for a 10% dropout and refusal rate, we planned to recruit 70 participants per group. In this study, the group with the minimum sample size was the urgency UI group, with 71 participants.

### Data Analysis

The data were analyzed using IBM SPSS Statistics version 22.0 (Armonk, NY, USA). Categorical variables are presented as frequencies and proportions and were compared via the Chi-squared test. Continuous variables are presented as the means and standard deviations and were subjected to univariate analysis of variance. Multivariate regression analysis was used to adjust for potential confounding factors related to symptom severity across different subtypes of UI, and subgroup analyses were further performed on the basis of the severity of UI symptoms. Statistical significance was predetermined at a *p* value lower than 0.05.

## Results

### Basic Clinical Characteristics of Participants with Various Subtypes of Urinary Incontinence

**Fig. 1:**
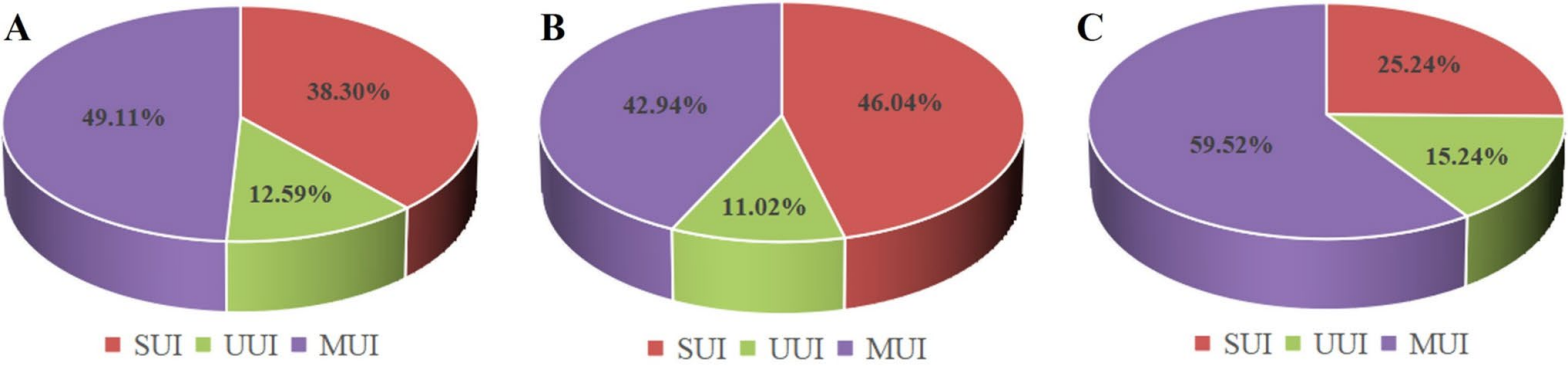
The constituent ratio of various subtypes of urinary incontinence among women seeking weight loss. **A** All participants. **B** Participants with mild symptoms. **C** Participants with moderate or severe symptoms

In total, 2,103 surveys were disseminated, with 1,693 respondents, representing an 80.5% response rate. After 488 subjects were eliminated based on the exclusion criteria, 564 out of 1,205 individuals (46.8%) were reported to present with UI symptoms. The mean age and BMI of the 564 participants were 36.7 years (range: 18–67) and 29.9 kg/m^2^ (range: 25–46.4) respectively. Among the 564 patients with UI, the proportions of patients with stress UI, urgency UI, and mixed UI were 216 (38.3%), 71 (12.6%), and 277 (49.1%) respectively (Fig. 1A). We subsequently compared the baseline clinical characteristics of participants with different subtypes of UI. As displayed in Table 1, except for the ICIQ-UI-SF (5.4 ± 3.0, 6.8 ± 3.8, and 6.9 ± 3.9 respectively), no significant differences were observed among the three groups at baseline.

**Table 1:** Comparison of basic clinical characteristics among women seeking weight loss with varying subtypes of urinary incontinence

Study characteristics	SUI (*n* = 216)	UUI (*n* = 71)	MUI (*n* = 277)	Statistic	*p*
Age (years)	36.7 ± 7.9	36.3 ± 8.6	36.7 ± 9	0.066	0.936
Body mass index (kg/m^2^)	30.1 ± 4.2	30.1 ± 4.6	29.7 ± 4.1	0.559	0.572
Parity	0.7 ± 0.7	0.7 ± 0.7	0.8 ± 0.7	0.743	0.476
History of vaginal delivery	70 (32.4%)	25 (35.2%)	109 (39.4%)	2.566	0.277
History of instrumental delivery	8 (3.7%)	4 (5.6%)	12 (4.3%)	0.496	0.78
History of cesarean delivery	70 (32.4%)	18 (25.4%)	89 (32.1%)	1.376	0.503
Menopause	22 (10.2%)	6 (8.5%)	33 (11.9%)	0.847	0.655
Smoker	17 (7.9%)	3 (4.2%)	22 (7.9%)	1.224	0.542
Asthma	8 (3.7%)	8 (11.3%)	15 (5.4%)	5.893	0.053
Allergic rhinitis	76 (35.2%)	21 (29.6%)	74 (26.7%)	4.143	0.126
Previous abdominal/pelvic surgery	82 (38%)	25 (35.2%)	112 (40.4%)	0.76	0.684
Metabolic syndrome	29 (13.4%)	10 (14.1%)	36 (13%)	0.063	0.969
Diabetes	26 (12%)	13 (18.3%)	34 (12.2%)	2.082	0.353
Dyslipidemia	41 (19%)	10 (14.1%)	61 (22%)	2.406	0.3
Hypertension	27 (12.5%)	11 (15.5%)	41 (14.8%)	0.683	0.711
Pelvic floor muscle training					
None	181 (83.8%)	59 (83.1%)	226 (81.6%)	0.425	0.809
Occasionally	35 (16.2%)	12 (16.9%)	51 (18.4%)		
Sexual frequency				4.123	0.39
None or occasionally	154 (71.3%)	52 (73.2%)	191 (69%)		
< 2 per week	55 (25.5%)	15 (21.1%)	66 (23.8%)		
≥ 2 per week	7 (3.2%)	4 (5.6%)	20 (7.2%)		
Toilet method				0.003	0.999
Squatting	27 (12.5%)	9 (12.7%)	35 (12.6%)		
Sitting	189 (87.5%)	62 (87.3%)	242 (87.4%)		
Occupation mode				1.157	0.561
Brainwork mainly	202 (93.5%)	66 (93%)	252 (91%)		
Physical work mainly	14 (6.5%)	5 (7%)	25 (9%)		
Physical labor				7.872	0.096
Light	172 (79.6%)	58 (81.7%)	196 (70.8%)		
Moderate	43 (19.9%)	13 (18.3%)	77 (27.8%)		
Heavy	1 (0.5%)	0	4 (1.4%)		
Educational background				2.307	0.679
High school and below	11 (5.1%)	4 (5.6%)	9 (3.2%)		
Undergraduate and Junior college	145 (67.1%)	47 (66.2%)	199 (71.8%)		
Graduate student	60 (27.8%)	20 (28.2%)	69 (24.9%)		
ICIQ-UI-SF	5.4 ± 3	6.8 ± 3.8*	6.9 ± 3.9**	12.258	< 0.001

*SUI* stress urinary incontinence, *UUI* urgency urinary incontinence, *MUI* mixed urinary incontinence, *ICIQ-UI-SF* International Consultation on Incontinence Modular Questionnaire-Urinary Incontinence Short Form

Compared with the SUI group, *p < 0.05, **p < 0.001

### The Constituent Ratios of Various Subtypes of Urinary Incontinence Stratified by Symptom Severity

Furthermore, a subgroup analysis was conducted on the basis of the severity of UI symptoms. Among the 354 participants with mild UI, the distributions of stress UI, urgency UI, and mixed UI were 163 (46%), 39 (11%), and 152 (43%) respectively (Fig. 1B). Moreover, among the 210 participants with moderate or severe UI, the proportions of patients with stress UI, urgency UI, and mixed UI were 53 (25.2%), 32 (15.2%), and 125 (59.5%) respectively (Fig. 1C).

### The Impact of Various Subtypes of Urinary Incontinence on Health-Related Quality of Life and Sexual Function

As shown in Table 2, regarding the UI-specific QoL evaluated by the UDI-6 and IIQ-7, the impact of the mixed UI was noticeably greater than that of the stress UI and urgency UI. In terms of the sexual function evaluated by the PISQ-12 and the general QoL evaluated by the EQ-5D-5L, the effect of the mixed UI was significantly greater than that of the stress UI. However, after controlling for the severity of UI, we found that the impact of mixed UI remained significantly greater than that of stress UI and urgency UI in terms of UI-specific QoL, whereas there was no significant difference among the three groups in terms of sexual function and general QoL.

**Table 2:** The impact of various subtypes of urinary incontinence on health-related quality of life and sexual function among women seeking weight loss

Questionnaires	SUI (*n* = 216)	UUI (*n* = 71)	MUI (*n* = 277)	*p*	Adj-P
UDI-6	2.2 ± 1.9***	2.3 ± 1.9***	4 ± 3.1	< 0.001	< 0.001
IIQ-7	1.6 ± 2.4***	2.1 ± 3.8*	3.4 ± 3.8	< 0.001	< 0.001
PISQ-12^a^	34.7 ± 3.3*	34.6 ± 3.8	33.9 ± 3.5	0.089	0.417
Behavioral/emotive	9.8 ± 1.1*	9.9 ± 1.1	9.5 ± 1.2	0.049	0.333
Physical	16.7 ± 1.5*	16.6 ± 2	16.3 ± 1.9	0.107	0.458
Partner-related	8.6 ± 1.2	8.4 ± 1.3	8.3 ± 1.1	0.096	0.451
EQ-5D-5L					
Mobility	0.998 ± 0.015	0.996 ± 0.022	0.996 ± 0.017	0.514	0.423
No problems	210 (97.2%)	68 (95.8%)	262 (94.6%)		
Slight problems	5 (2.3%)	2 (2.8%)	14 (5.1%)		
Moderate problems	1 (0.5%)	1 (1.4%)	1 (0.4%)		
Self-care	0.1 ± 0.003	0.999 ± 0.006	0.999 ± 0.006	0.408	0.327
No problems	215 (99.5%)	70 (98.4%)	272 (98.2%)		
Slight problems	1 (0.5%)	1 (1.6%)	5 (1.8%)		
Usual activities	0.998 ± 0.012	0.994 ± 0.02	0.998 ± 0.01	0.042	0.061
No problems	210 (97.2%)	65 (91.5%)	269 (97.1%)		
Slight problems	4 (1.9%)	4 (5.6%)	7 (2.5%)		
Moderate problems	2 (0.9%)	2 (2.8%)	1 (0.4%)		
Pain/discomfort	0.979 ± 0.043	0.972 ± 0.051	0.974 ± 0.04	0.334	0.344
None	155 (71.8%)	46 (64.8%)	175 (63.2%)		
Slight	54 (25%)	21 (29.6%)	89 (32.1%)		
Moderate	3 (1.4%)	2 (2.8%)	11 (4%)		
Severe	4 (1.9%)	2 (2.8%)	2 (0.7%)		
Anxiety/depression	0.963 ± 0.043*	0.956 ± 0.047	0.954 ± 0.045	0.075	0.468
None	95 (44%)	26 (36.6%)	93 (33.6%)		
Slight	95 (44%)	35 (49.3%)	136 (49.1%)		
Moderate	23 (10.6%)	8 (11.3%)	43 (15.5%)		
Severe	3 (1.4%)	2 (2.8%)	5 (1.8%)		
Mean utility value	0.988 ± 0.015*	0.984 ± 0.022	0.984 ± 0.015	0.057	0.468
EQ-VAS	74 ± 14.4	71.7 ± 15	72.1 ± 16.6	0.303	0.557

*SUI* stress urinary incontinence, *UUI* urgency urinary incontinence, *MUI* mixed urinary incontinence, *UDI-6* Urinary Distress Inventory 6, *UIQ-7* Urinary Incontinence Quality of Life Scale 7, *PISQ-12* Short-form Prolapse Incontinence Sexual Questionnaire, *EQ-5D-5L* European Quality of Life-5 Dimensions 5-Level questionnaire, *EQ-VAS* associated visual analogue scale of the EQ-5D-5L, *Adj* adjusted by the International Consultation on Incontinence Modular Questionnaire-Urinary Incontinence Short Form scores

Compared with the MUI group, *p < 0.05, **p < 0.01, ***p < 0.001

^a^152, 44, and 162 patients respectively completed the PISQ-12

### The Impact of Various Subtypes of Urinary Incontinence on Health-Related Quality of Life and Sexual Function Stratified by Symptom Severity

**Fig. 2:**
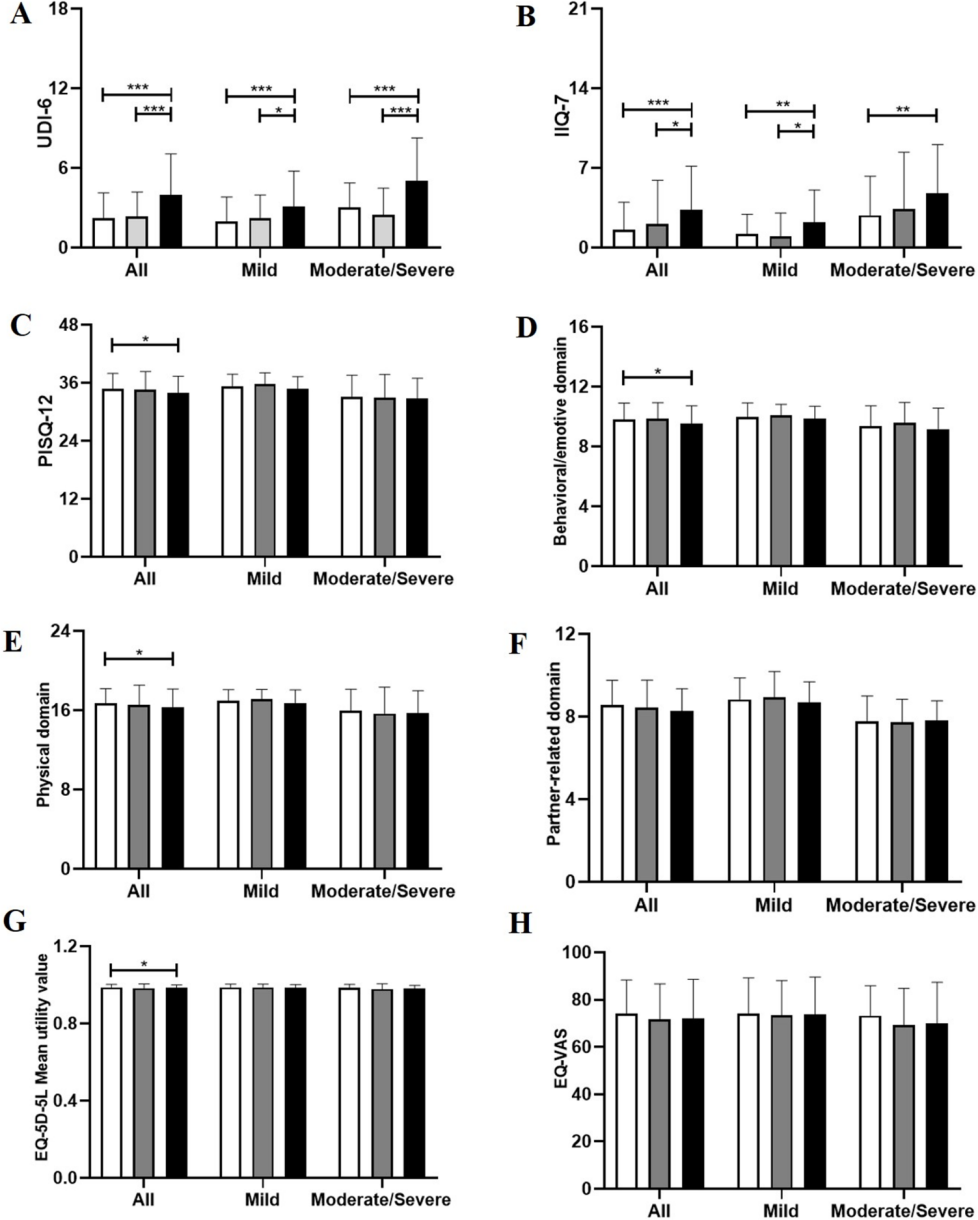
The impact of various subtypes of urinary incontinence on health-related quality of life and sexual function stratified by symptom severity. White box: stress urinary incontinence, gray box: urgency urinary incontinence, black box: mixed urinary incontinence. The number of patients who completed the PISQ-12 in the three groups was 152 (124 mild and 28 moderate/severe), 44 (30 mild and 14 moderate/severe), and 162 (96 mild and 66 moderate/severe). **A** *UDI-6* Urinary Distress Inventory 6, **B** *UIQ-7* Urinary Incontinence Quality of Life Scale 7, **C** *PISQ-12* Prolapse Incontinence Sexual Questionnaire short form, **D**–**F** the three domains of PISQ-12, **G** *EQ-5D-5L* European Quality of Life-5 Dimensions 5-Level, **H** *EQ-VAS* the associated visual analogue scale of the EQ-5D-5L. * *p*  < 0.05, ** *p*  < 0.01, *** *p*  < 0.001

We subsequently performed a stratified analysis on the basis of the severity of UI (Fig. 2). Upon stratification, no differences in baseline characteristics were observed among the three groups (Supplementary Tables 1 and 2). Consistent with the aforementioned results, among individuals with mild and moderate/severe UI, mixed UI had the greatest impact on UI-specific QoL, whereas no differences were found in sexual function or general QoL among the three groups.

## Discussion

Our study investigated the constituent ratios of different subtypes of UI among women with overweight or obesity and the differences in their QoL and sexual function. We demonstrated variations in the constituent ratios of UI subtypes among patients with different degrees of UI. The severity of UI was greater in urgency UI and mixed UI than in stress UI, with varying subtype ratios observed at different severity levels: stress UI was greatest in mild cases (46.0%), and the mixed UI was greatest in moderate or severe cases (59.5%). Mixed UI had the most pronounced effect on QoL and sexual function. However, after controlling for UI severity, the mixed UI had a significantly greater impact on the UI-specific QoL, as evaluated by the UDI-6 and IIQ-7, whereas no differences were identified among the three groups regarding the general QoL, as evaluated by the EQ-5D-5L, or sexual function, as evaluated by the PISQ-12. These findings provide valuable insights into the characteristics of UI among women with overweight or obesity, highlighting the necessity of developing personalized treatment strategies for different subtypes of UI and offering scientific evidence for clinical treatment. Future research is needed to further investigate how lifestyle changes, medication, or surgical interventions can improve UI symptoms in women with overweight or obesity, thereby increasing their QoL.

Overweight and obesity have been established as important risk factors for UI, and current guidelines [4] advocate for multidisciplinary interventions encompassing nutrition and gynecological care for this high-risk population. However, current surveys still reveal a glaring lack of public knowledge regarding the heightened susceptibility to PFD conferred by obesity [19]. Previous research [6, 20] has demonstrated that the severity of UI symptoms increases with increased BMI and prolonged periods of obesity, underscoring the importance of lifelong weight management strategies. Currently, public awareness and physician engagement with this demographic information are still relatively inadequate, with a dearth of more personalized studies. A previous Brazilian study [12] included 221 individuals with obesity, 118 of whom (53.4%) experienced UI episodes: mixed UI, stress UI, and urgency UI were reported by 52.5% (62), 33.9% (40), and 13.6% (16) of the participants respectively, which aligns with our findings. However, when accounting for the severity of UI, there are stark discrepancies in the distribution of UI subtypes, with stress UI, urgency UI, and mixed UI accounting for 46%, 11%, and 43% respectively of the mild cases, and 25.2%, 15.2%, and 59.5% respectively of the moderate or severe cases.

Several previous studies have investigated the impact of UI subtypes on UI-specific QoL, and their findings align with ours, indicating that mixed UI may have the most detrimental effects [10, 21, 22]. However, in this study, the scores of patients on the UDI-6 and IIQ-7 were significantly lower than those reported in previous studies, which could be attributed to patient recruitment being conducted among those seeking medical weight loss rather than those seeking medical treatment owing to symptoms of UI. QoL can be assessed via both general and disease-specific tools. Disease-specific questionnaires effectively capture UI-related changes but may lack comprehensiveness. Women with overweight and obesity often face multifaceted QoL impacts, warranting a holistic assessment. Recent research has underscored the efficacy of the EQ-5D-5L in assessing QoL among individuals with UI, demonstrating robust construct validity, responsiveness, and reliability [23, 24]. However, current research lacks insights into the effects of different UI types on general QoL among women with overweight or obesity. In our study, the mixed UI had a significant negative impact on the general QoL, particularly in the anxiety/depression domain. However, after accounting for symptom severity, no significant differences emerged among the three groups regarding the general QoL.

The existing results examining the impact of different subtypes of UI on sexual function remain controversial [9, 10, 25]. The use of diverse assessment methods and variations in the definitions and classification of UI across different studies may contribute to these discordant findings. However, studies focused on the prevalence and impact of different UI subtypes among women with overweight or obesity are relatively rare. Our findings address this knowledge gap by revealing that mixed UI has the most significant negative impact on sexual function, encompassing both the behavioral/emotional and physical domains. However, the apparent greater impact of mixed UI on sexual function may be due to the greater severity of incontinence experienced by mixed UI patients. After controlling for UI severity, no significant differences were observed among the three groups. These findings could be attributed to several factors common to all forms of incontinence, such as the necessity of using a pad during intercourse, concerns about odor, and fears of urine leakage.

Our research holds clinical significance for several reasons. First, we employed all validated questionnaires, ensuring the reliability and applicability of the collected data. Second, we conducted a comprehensive evaluation of the various influences of UI subtypes on sexual function and QoL among women with overweight or obesity, providing a multidimensional assessment of the impact of different subtypes of UI on patients. However, it is crucial to acknowledge the limitations of our study. First, owing to its cross-sectional design, causality cannot be established, which limits our ability to determine the temporal relationship between UI subtypes and outcomes. Despite our efforts to account for potential confounding factors, the presence of some unmeasured confounders that could influence our results cannot be ruled out. Second, reliance on self-reported measures may lead to response bias and other errors, potentially compromising the validity of the results. Despite some measures taken to enhance data accuracy, the accuracy of the severity assessment of UI may still be compromised by recall bias or social desirability bias. Third, by limiting our sample to women actively seeking weight loss, the generalizability of the study’s findings, particularly those not seeking weight loss, is restricted. Last, although our center receives patients from a wide geographical area and recruitment was conducted consecutively, the inherent selection bias of a single-center study remains. Furthermore, despite the broad range in both patient age and BMI, the limited sample size prevented us from conducting further stratified analyses. In future investigations, to circumvent these limitations and derive more credible and rigorous conclusions, multicenter studies encompassing larger sample sizes and employing a prospective design will be essential.

## Conclusion

This study demonstrated variations in the constituent ratios of UI subtypes related to the severity of UI and the effects of various UI subtypes on QoL and sexual function among women seeking weight loss. Notably, mixed UI demonstrated the most severe symptoms and the most unfavorable impact, particularly as assessed by UI-specific QoL questionnaires. These findings provide valuable insights into the characteristics of UI in women with overweight or obesity, underscoring the need for personalized treatment strategies for various UI subtypes and providing a scientific foundation for clinical interventions. Future research should explore the potential improvements in UI symptoms among women with overweight or obesity through lifestyle changes, medications, or surgical procedures, ultimately enhancing their QoL.

## Supplementary Information

Below is the link to the electronic supplementary material.

Supplementary file1 (DOCX 29 KB)

Supplementary file2 (DOCX 23 KB)

## Data Availability

Data used for the current study are available from the corresponding author upon reasonable request.
