# Supplementary material for: Effects of Urinary Incontinence Subtypes on Quality of Life and Sexual Function among Women Seeking Weight Loss
Source: Int Urogynecol J. 2024 Nov 21;36(2):381–9. doi: 10.1007/s00192-024-05977-z (PMC11850504; doi:10.1007/s00192-024-05977-z)
Supplement: Supplementary file 2 — Supplementary file2 (DOCX 23 KB) [file 192_2024_5977_MOESM2_ESM.docx]

| **Study characteristics** | | **SUI (n=163)** | **UUI (n=39)** | **MUI (n=152)** | **Statistic** | **P** |
| --- | --- | --- | --- | --- | --- | --- |
| **Age (years)** | | 36.3±7.8 | 34.8±8.1 | 35.6±8.3 | 0.674 | 0.511 |
| **Body mass index (kg/m^2^)** | | 30±4 | 29.7±3.9 | 29.4±4.2 | 0.85 | 0.428 |
| **Parity** | | 0.7±0.7 | 0.5±0.6 | 0.7±0.7 | 1.94 | 0.145 |
| **History of vaginal delivery** | | 47(28.8%) | 11(28.2%) | 58(38.2%) | 3.518 | 0.172 |
| **History of instrument delivery** | | 4(2.5%) | 3(7.7%) | 7(4.6%) | 2.57 | 0.277 |
| **History of cesarean delivery** | | 52(31.9%) | 7(17.9%) | 42(27.6%) | 3.110 | 0.211 |
| **Menopause** | | 15(7.4%) | 4(10.3%) | 13(8.6%) |  |  |
| **Smoker** | | 12(6.7%) | 2(5.1%) | 11(7.2%) | 1.274 | 0.529 |
| **Asthma** | | 6(3.7%) | 4(10.3%) | 5(3.3%) | 3.943 | 0.139 |
| **Allergic rhinitis** | | 57(35.0%) | 12(30.8%) | 43(28.3%) | 1.638 | 0.441 |
| **Previous abdominal/pelvic surgery** | | 59(36.2%) | 10(25.6%) | 54(35.5%) | 1.618 | 0.445 |
| **Metabolic syndrome** | | 16(9.8%) | 7(17.9%) | 18(11.8%) | 2.05 | 0.359 |
|  | ***Diabetes*** | 17(10.4%) | 6(15.4%) | 17(11.2%) | 0.774 | 0.679 |
|  | ***Dyslipidemia*** | 24(14.7%) | 6(15.4%) | 29(19.1%) | 1.126 | 0.57 |
|  | ***Hypertension*** | 17(10.4%) | 6(15.4%) | 20(13.2%) | 0.979 | 0.613 |
| **Pelvic floor muscle training** | |  |  |  | 0.791 | 0.673 |
|  | ***None*** | 134(82.2%) | 31(79.5%) | 129(84.9%) |  |  |
|  | ***Occasionally*** | 29(17.8%) | 8(20.5%) | 23(15.1%) |  |  |
| **Sexual frequency** | |  |  |  | 3.664 | 0.453 |
|  | ***None or occasionally*** | 112(68.7%) | 28(71.8%) | 106(69.7%) |  |  |
|  | ***< 2 per week*** | 46(28.2%) | 9(23.1%) | 35(23.0%) |  |  |
|  | ***≥ 2 per week*** | 5(3.1%) | 2(5.1%) | 11(7.2%) |  |  |
| **Toilet way** | |  |  |  | 1.312 | 0.519 |
|  | ***Squatting*** | 20(12.3%) | 7(17.9%) | 17(11.2%) |  |  |
|  | ***Sitting*** | 143(87.7%) | 32(82.1%) | 135(88.8%) |  |  |
| **Occupation mode** | |  |  |  | 0.139 | 0.933 |
|  | ***Brainwork mainly*** | 152(93.3%) | 37(94.9%) | 142(93.4%) |  |  |
|  | ***Physical work mainly*** | 11(6.7%) | 2(5.1%) | 10(6.6%) |  |  |
| **Physical labor** | |  |  |  | 4.354 | 0.36 |
|  | ***Light*** | 129(79.1%) | 33(84.6%) | 109(71.7%) |  |  |
|  | ***Moderate*** | 33(20.2%) | 6(15.4%) | 41(27.0%) |  |  |
|  | ***Heavy*** | 1(0.6%) | 0 | 2(1.3%) |  |  |
| **Educational background** | |  |  |  | 0.729 | 0.948 |
|  | ***High school and below*** | 7(4.3%) | 2(5.1%) | 5(3.3%) |  |  |
|  | ***Undergraduate and Junior college*** | 113(69.3%) | 25(64.1%) | 106(69.7%) |  |  |
|  | ***Graduate student*** | 43(26.4%) | 12(30.8%) | 41(27.0%) |  |  |
|  | **ICIQ-UI-SF** | 4±1.4 | 4.2±1.3 | 4.4±1.5 | 0.21 | 0.811 |

**Supplementary Table 1 The basic clinical characteristics of various subtypes of urinary incontinence among women with mild symptoms.** SUI: stress urinary incontinence, UUI: urgency urinary incontinence, MUI: mixed urinary incontinence, ICIQ-UI-SF: International Consultation on Incontinence Modular Questionnaire-Urinary Incontinence Short Form.

**Supplementary Table 2 The basic clinical characteristics of various subtypes of urinary incontinence among women with moderate or severe symptoms.** SUI: stress urinary incontinence, UUI: urgency urinary incontinence, MUI: mixed urinary incontinence, ICIQ-UI-SF: International Consultation on Incontinence Modular Questionnaire-Urinary Incontinence Short Form.

| **Study characteristics** | | **SUI (n=53)** | **UUI (n=32)** | **MUI (n=125)** |  | **P** |
| --- | --- | --- | --- | --- | --- | --- |
| **Age (years)** | | 38.1±7.8 | 38.2±8.9 | 38±9.8 | 0.004 | 0.996 |
| **Body mass index (kg/m^2^)** | | 30.3±4.7 | 30.6±5.4 | 30.1±3.9 | 0.213 | 0.808 |
| **Parity** | | 0.9±0.8 | 0.9±0.8 | 0.9±0.7 | 0.207 | 0.431 |
| **History of vaginal delivery** | | 23(43.4%) | 14(43.8%) | 51(40.8%) | 0.156 | 0.925 |
| **History of instrument delivery** | | 4(7.5%) | 1(3.1%) | 5(4.0%) | 1.256 | 0.534 |
| **History of cesarean delivery** | | 18(34.0%) | 11(34.4%) | 47(37.6%) | 0.267 | 0.875 |
| **Menopause** | | 7(13.2%) | 2(6.3%) | 20(16.0%) | 2.056 | 0.358 |
| **Smoker** | | 5(9.4%) | 1(3.1%) | 11(8.8%) | 0.119 | 0.942 |
| **Asthma** | | 2(3.8%) | 4(12.5%) | 10(8.0%) | 2.222 | 0.329 |
| **Allergic rhinitis** | | 19(35.8%) | 9(28.1%) | 31(24.8%) | 2.249 | 0.325 |
| **Previous abdominal/pelvic surgery** | | 23(43.4%) | 15(46.9%) | 58(46.4%) | 0.156 | 0.925 |
| **Metabolic syndrome** | | 13(24.5%) | 3(9.4%) | 18(14.4%) | 4.106 | 0.128 |
|  | ***Diabetes*** | 11(20.8%) | 4(12.5%) | 18(14.4%) | 1.429 | 0.489 |
|  | ***Dyslipidemia*** | 17(32.1%) | 4(12.5%) | 32(25.6%) | 4.074 | 0.13 |
|  | ***Hypertension*** | 10(18.9%) | 5(15.6%) | 21(16.8%) | 0.173 | 0.917 |
| **Pelvic floor muscle training** | |  |  |  | 3.88 | 0.144 |
|  | ***None*** | 47(88.9%) | 28(82.6%) | 97(79.5%) |  |  |
|  | ***Occasionally*** | 6(11.3%) | 4(12.5%) | 28(22.4%) |  |  |
| **Sexual frequency** | |  |  |  | 2.621 | 0.623 |
|  | ***None or occasionally*** | 42(79.2%) | 24(75.0%) | 85(68.0%) |  |  |
|  | ***< 2 per week*** | 9(17.0%) | 6(18.8%) | 31(24.8%) |  |  |
|  | ***≥ 2 per week*** | 2(3.8%) | 2(6.3%) | 9(7.2%) |  |  |
| **Toilet way** | |  |  |  | 1.518 | 0.468 |
|  | ***Squatting*** | 7(13.2%) | 2(6.3%) | 18(14.4%) |  |  |
|  | ***Sitting*** | 46(86.8%) | 30(93.7%) | 107(85.6%) |  |  |
| **Occupation mode** | |  |  |  | 1.678 | 0.432 |
|  | ***Brainwork mainly*** | 50(94.3%) | 29(90.6%) | 110(88.0%) |  |  |
|  | ***Physical work mainly*** | 3(5.7%) | 3(9.4%) | 15(12.0%) |  |  |
| **Physical labor** | |  |  |  | 3.75 | 0.441 |
|  | ***Light*** | 43(81.1%) | 25(78.1%) | 87(69.6%) |  |  |
|  | ***Moderate*** | 10(18.9%) | 7(21.9%) | 36(28.8%) |  |  |
|  | ***Heavy*** | 0 | 0 | 2(1.6%) |  |  |
| **Educational background** | |  |  |  | 4.088 | 0.394 |
|  | ***High school and below*** | 4(7.5%) | 2(6.3%) | 4(3.2%) |  |  |
|  | ***Undergraduate and Junior college*** | 32(60.4%) | 22(68.8%) | 93(74.4%) |  |  |
|  | ***Graduate student*** | 17(32.1%) | 8(25.0%) | 28(22.4%) |  |  |
|  | **ICIQ-UI-SF** | 9.5±2.7 | 10±3.4 | 10.4±3.1 | 1.397 | 0.25 |
